# Carotid artery intima-media thickness measurement in children with normal and increased body mass index: a comparison of three techniques

**DOI:** 10.1007/s00247-018-4144-6

**Published:** 2018-05-09

**Authors:** Ramy El Jalbout, Guy Cloutier, Marie-Hélène Roy Cardinal, Mélanie Henderson, Chantale Lapierre, Gilles Soulez, Josée Dubois

**Affiliations:** 1 Department of Medical Imaging, University of Montreal, Sainte-Justine University Health Center, 3175 Cote-Sainte-Catherine Road, Montreal, Quebec, H3T 1C5 Canada; 2 Laboratory of Biorheology and Medical Ultrasonics, University of Montreal Hospital Research Center (CRCHUM), Montreal, Quebec, Canada; 3 Department of Pediatrics, University of Montreal, Sainte-Justine University Health Center, Montreal, Quebec, Canada; 4 Department of Radiology, University of Montreal Hospital Center (CHUM), Montreal, Quebec, Canada

**Keywords:** Atherosclerosis, B-mode ultrasound, Carotid artery, Children, Intima-media thickness, Obesity, Radiofrequency echo tracking

## Abstract

**Background:**

Common carotid artery intima-media thickness is a marker of subclinical atherosclerosis. In children, increased intima-media thickness is associated with obesity and the risk of cardiovascular events in adulthood.

**Objective:**

To compare intima-media thickness measurements using B-mode ultrasound, radiofrequency (RF) echo tracking, and RF speckle probability distribution in children with normal and increased body mass index (BMI).

**Materials and methods:**

We prospectively measured intima-media thickness in 120 children randomly selected from two groups of a longitudinal cohort: normal BMI and increased BMI, defined by BMI ≥85th percentile for age and gender. We followed Mannheim recommendations. We used M’Ath-Std for automated B-mode imaging, M-line processing of RF signal amplitude for RF echo tracking, and RF signal segmentation and averaging using probability distributions defining image speckle. Statistical analysis included Wilcoxon and Mann-Whitney tests, and Pearson correlation coefficient and intra-class correlation coefficient (ICC).

**Results:**

Children were 10–13 years old (mean: 11.7 years); 61% were boys. The mean age was 11.4 years (range: 10.0–13.1 years) for the normal BMI group and 12.0 years (range: 10.1–13.5 years) for the increased BMI group. The normal BMI group included 58% boys and the increased BMI group 63% boys. RF echo tracking method was successful in 79 children as opposed to 114 for the B-mode method and all 120 for the probability distribution method. Techniques were weakly correlated: ICC=0.34 (95% confidence interval [CI]: 0.27–0.39). Intima-media thickness was significantly higher in the increased BMI than normal BMI group using the RF techniques and borderline for the B-mode technique. Mean differences between weight groups were: B-mode, 0.02 mm (95% CI: 0.00 to 0.04), *P*=0.05; RF echo tracking, 0.03 mm (95% CI: 0.01 to 0.05), *P*=0.01; and RF speckle probability distribution, 0.03 mm (95% CI: 0.01 to 0.05), *P*=0.002.

**Conclusion:**

Though techniques are not interchangeable, all showed increased intima-media thickness in children with increased BMI. RF echo tracking method had the lowest success rate at calculating intima-media thickness. For patient follow-up and cohort comparisons, the same technique should be used throughout.

## Introduction

Common carotid artery intima-media thickness is a reliable marker of subclinical atherosclerosis [1]. In children, increased intima-media thickness is associated with obesity and the risk of cardiovascular events in adulthood [1, 2]. The use of intima-media thickness in risk stratification, however, is limited by two factors. The first is a lack of standardization in the acquisition and analysis of intima-media thickness measures. The second is a lack of reproducibility and validated age- and gender-specific normative values against which to compare patient results. The Association for European Paediatric Cardiology (AEPC) recognized the need for standardized normative data in its recommendations for the measurement, analysis and interpretation of intima-media thickness in children [3].

There are two main methods for measuring common carotid artery intima-media thickness: ultrasound (US) B-mode images (manual, semiautomated or automated) and radiofrequency (RF) multiple M-line analysis (using echo tracking of RF amplitude). In adults, the two methods correlate well [4]. Automated B-mode imaging with dedicated software (M’Ath-Std) was validated for intima-media thickness measurement and showed excellent reproducibility in the PARC (Paroi Artérielle et Risque Cardiovasculaire) study [5]. In pediatrics, studies used either US [6] or echo tracking [7]. To our knowledge, there are no studies comparing B-mode and RF in children and few published normative values using either technique [3, 6, 7]. B-mode US has nonetheless demonstrated increased intima-media thickness in obese children [8].

The objective of this study was therefore to compare common carotid artery intima-media thickness using B-mode US, RF echo tracking, and segmentation of the vessel wall using RF speckle probability distribution in children with normal and increased body mass index (BMI).

## Materials and methods

### Study design and patient population

We randomly selected 120 children from two groups of the Quebec Adipose and Lifestyle Investigation in Youth (QUALITY) prospective cohort of 564 children: a normal BMI group and an increased BMI group, as defined by BMI ≥85th percentile for age and gender, 2 years after enrollment in the cohort between 2005 and 2011. The study was approved by the Ethics Boards of the Quebec Heart and Lung Institute and Sainte-Justine University Hospital. Written informed assent and consent were obtained from all participants and their parents, respectively. Inclusion criteria were Caucasian origin, age 8–10 years at time of entry into the original cohort, healthy, and having at least one obese parent (defined by BMI ≥30 kg/m^2^ or an elevated waist circumference (>102 cm in males and >88 cm in females) [9]. Exclusion criteria included being a pregnant or breastfeeding mother, families planning to move, and children on a restricted diet or taking anti-hypertensive drugs or corticosteroids [10, 11].

The independent variables included BMI, age and gender. All information was collected during the same visit. Weight was measured twice on an electronic scale to the nearest 0.1 kg. Height was measured twice to the nearest 0.1 cm. BMI was calculated according to the formula weight (kg)/height (m)^2^. We defined overweight and obesity according to the Centers for Disease Control age- and gender-adjusted z-score for weight and height [12].

### Image acquisition

Primary end points were intima-media thickness measured by B-mode, RF echo tracking and RF speckle probability distribution. Images were acquired according to the Mannheim recommendations [13]. Participants were in the supine position with their head tilted 45° to the opposite side. We measured the far wall of the common carotid artery at 1 cm from the carotid bifurcation. A high frequency linear US probe was positioned longitudinally perpendicular to the vessel wall. The software, whether for the B-mode measurement or for the RF echo tracking measurement, calculates the intima-media thickness automatically along the indicated segment.

### B-mode intima-media thickness

**Fig. 1:**
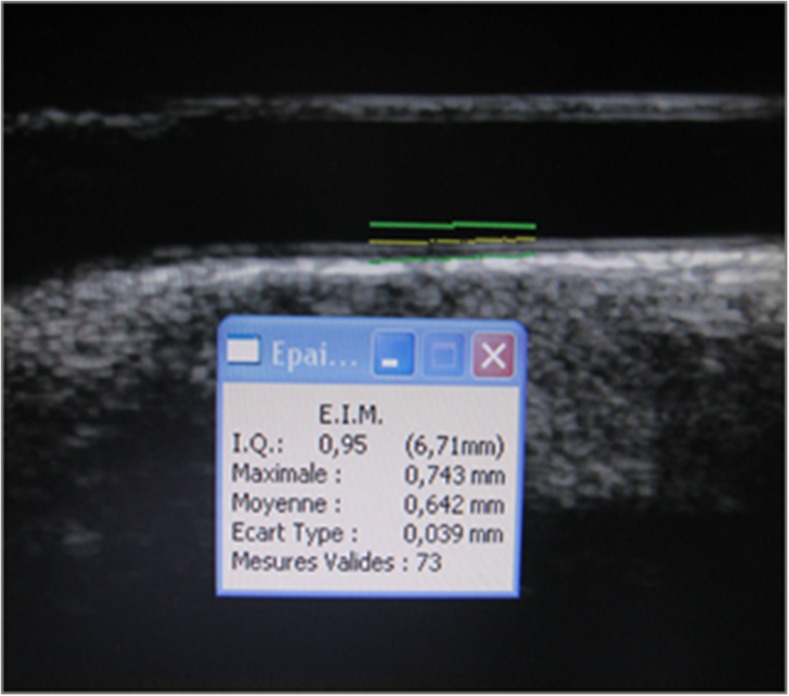
B-mode longitudinal US image of the common carotid artery of a 12-year-old boy shows the line drawn by the technologist next to the intima-media-lumen interface indicating the location of the automatic measurement by the M’Ath-Std system software. The software automatically gave the intima-media thickness value averaged over the 1-cm segment of the vessel and gave the quality index (we aimed an index superior to 0.5 as explained in text)

B-mode images were obtained using an ATL 5000 HDI US unit (Advanced Technology Laboratories, Bothell, WA) equipped with a 5.5–12.5 MHz probe. Focus depth was set around 25 mm, with a frame rate of 25 per s and gain settings adjusted optimally to facilitate edge detection. A US technologist with 10 years of experience scanned the child and saved a static longitudinal image of the common carotid artery that shows best the intima-media-lumen interface on gray-scale image. The 2-D image is instantaneously transferred to a computer with M’Ath-Std software (Metris, Argenteuil, France). The technologist then drew a 1-cm-long line next to the intima-media-lumen interface (Fig. 1). Automated computerized intima-media thickness is then generated by the software [14, 15]. We sought a quality index >0.5 for each measure indicating that more than 50% of the measurements along the 1-cm-long line were used to calculate the final averaged intima-media thickness. An average of two values was obtained during optimal resolution, independent of cardiac cycle.

### RF echo tracking intima-media thickness

**Fig. 2:**
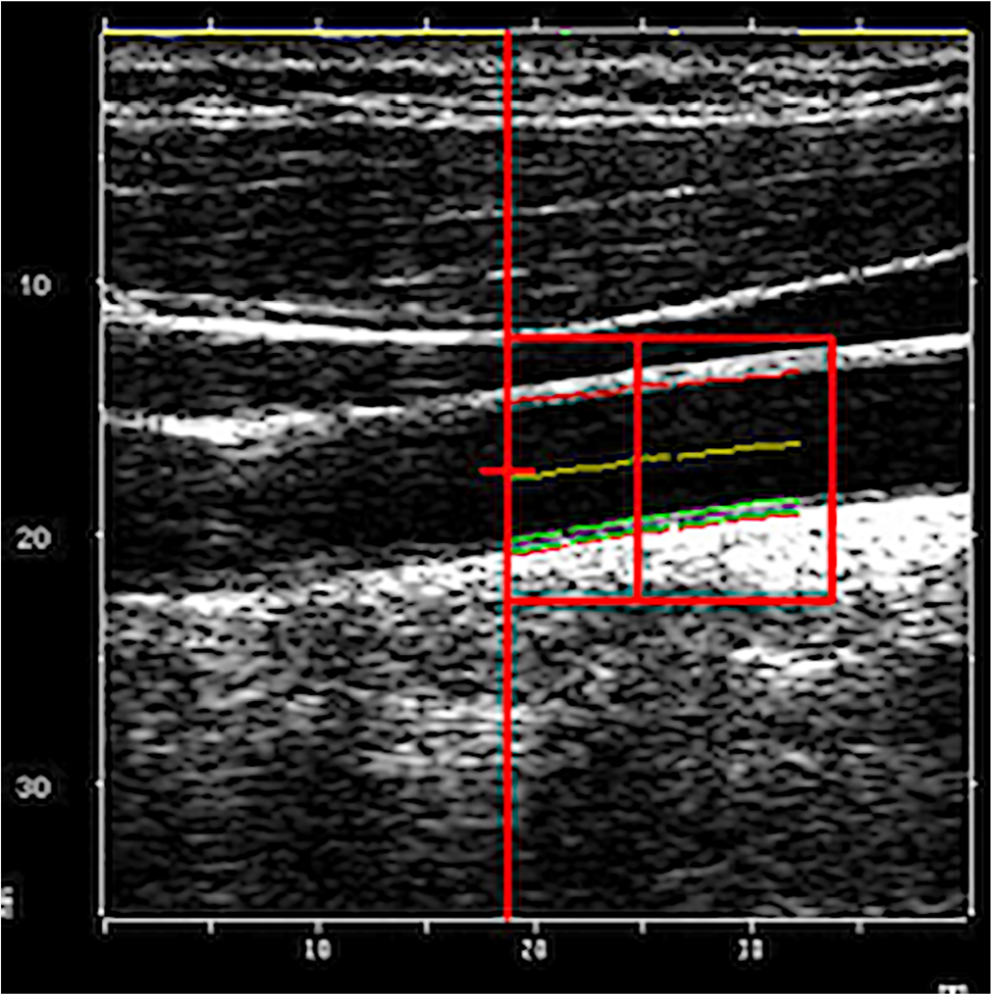
Radiofrequency echo tracking static image of an 11-year-old girl shows the location to measure the intima-media thickness (green) on the far vessel wall indicated by the technologist. The software automatically gave individual intima-media thickness values during the entire cardiac cycle depending on the lumen diameter as well as the averaged final value

We used a linear array L10–5 40-mm US transducer (model 410,503) on the MyLab 70 platform (ESAOTE) equipped with an ART.LAB platform (Pie Medical Imaging BV, Maastricht, The Netherlands). A 2-5 cardiac cycles long RF-digitized data stream is recorded by the technologist who then indicates a region of interest along the intima-media-lumen interface. Automated intima-media thickness is then obtained by calculating RF US signals received along a single line of observation (M-line processing) with time-averaging (using an electrocardiogram [ECG]) and motion compensation algorithms [16]. An average of two such intima-media thickness values was obtained (Fig. 2).

### RF speckle probability distribution intima-media thickness

**Fig. 3:**
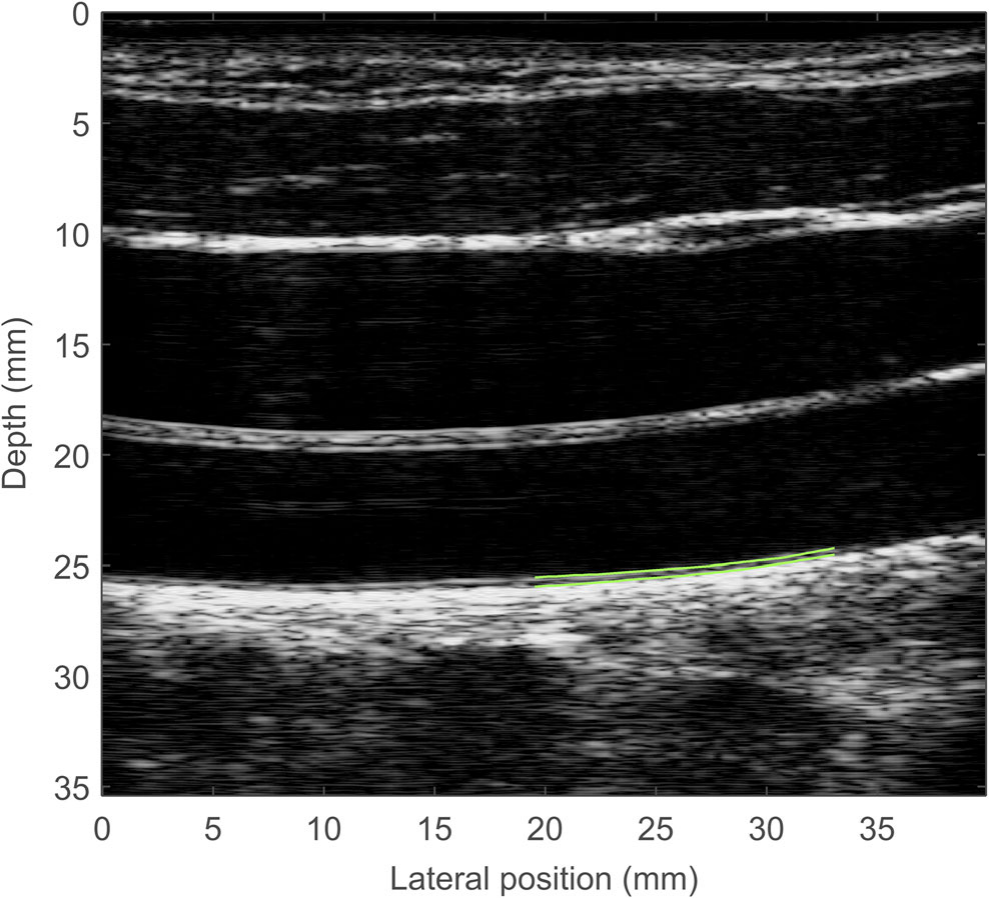
A static image of the common carotid artery of a 12-year-old boy demonstrates the radiofrequency speckle probability distribution technique taken from a video sequence on which automatically computed contours of the intima-media borders (green) with the lumen and adventitia are shown. The segmentation is manually initialized on a single frame (not shown). Both contours are then automatically traced on all subsequent frames of the 2–5 cardiac cycle-long video sequence. The intima-media thickness is the average (over all frames) of the mean distance between the two segmented contours

The same RF-digitized data stream (cine-loop acquisition of 2 to 5 cardiac cycles including 250–300 images) was post-processed to obtain an intima-media thickness using a different semiautomatic algorithm. This algorithm was implemented on a dedicated platform (ORS Visual; Object Research Systems Inc., Montreal, Canada). A research assistant (M.-H. R. C.) with 8 years of experience blinded to the results of B mode and RF echo tracking manually initialized the segmentation on one image of the data stream by drawing two contours: the lumen/intima-media and the intima-media/adventitia interfaces. The software automatically computed these two interfaces on all subsequent frames (Fig. 3). The intima-media thickness was the average of all segmented frames of the cine-loop of the mean distance between the two segmented interfaces. The segmentation of the vessel wall was based on the method described by Destrempes et al. [17, 18]. During the segmentation process, the initial contours are refined and tracked over the whole video sequence. For this purpose, a Bayesian segmentation model was used. In this model, the echogenicity of the intima-media layer, the lumen and the adventitia are modeled by mixtures of Nakagami distributions and the motion of the vessel wall is estimated with optical flow. An average of systolic and diastolic intima-media thickness of all subsegments is obtained.

### Statistical analysis

We did a descriptive analysis of the study population, checked for normality of the data distribution using the Shapiro-Wilk test, and used the Wilcoxon test for paired samples to compare intima-media thickness obtained by each of the three techniques. The intra-class correlation coefficient (ICC) was used to measure correlation. We plotted Bland-Altman and regression graphs to verify the ranges of agreement. We checked for the correlation between intima-media thickness and age using the Pearson correlation. We used the Mann-Whitney *U* test for the difference between children with normal BMI and those with increased BMI. Analysis was done using MedCalc statistical software version 17.9.7 (Ostend, Belgium).

## Results

**Fig. 4:**
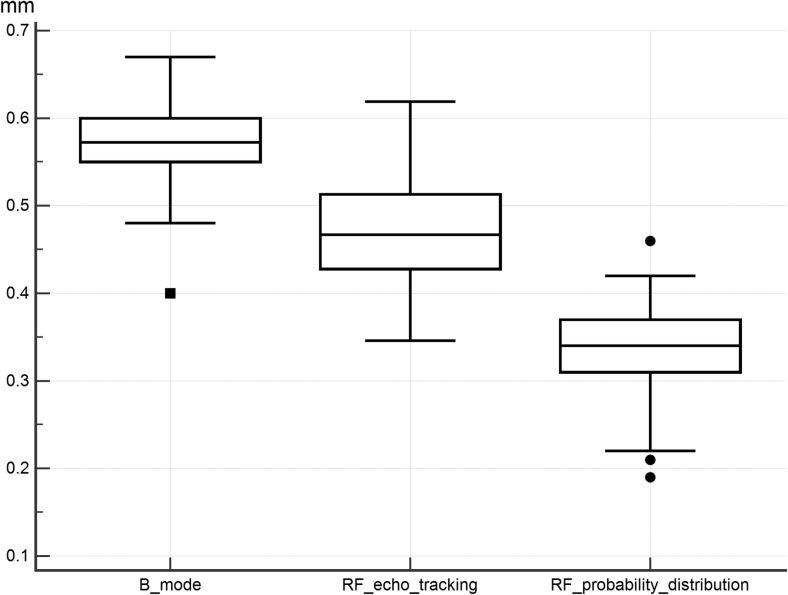
Box and whisker plot of mean intima-media thickness: B-mode: 0.56+/−0.004 mm (95% confidence interval [CI]: 0.55–0.57), radiofrequency echo tracking: 0.47+/−0.006 mm (95% CI: 0.46–0.48) and radiofrequency probability distribution: 0.34+/−0.005 mm (95% CI: 0.33–0.35)

The age range of the 120 randomly selected children was 10 to 13 years including 73 boys. The mean age was 11.4 years (95% confidence interval [CI]: 11.2–11.6) for the normal BMI group and 12.0 years (95% CI: 11.8–12.2) for the increased BMI group. In the normal BMI group, 58% were boys, and in the increased BMI group 63% were boys. The two groups were significantly different with respect to BMI (mean z-score=−0.73 (95% CI: -0.92 to-0.54) for the normal BMI group and 1.96 (95% CI: 1.83–2.08) for the increased BMI group *(P*<0.001). The age was statistically different (*P*<0.001). Gender ratios did not differ significantly between the groups (*P*=0.7). Although we measured intima-media thickness on all children, data for RF echo tracking were often not available as the software could not automatically detect the intima-media-lumen interface mainly due to artifacts. We could not call the children back as the present analysis was started in 2016, at least 5 years after data collection. Final analysis included 114 children for B-mode, 79 for RF echo tracking and 120 for RF speckle probability distribution. Therefore, for the 74 children who had all three measurements available, intima-media thickness differed significantly depending on technique. Mean intima-media thicknesses are shown in Fig. 4.

**Fig. 5:**
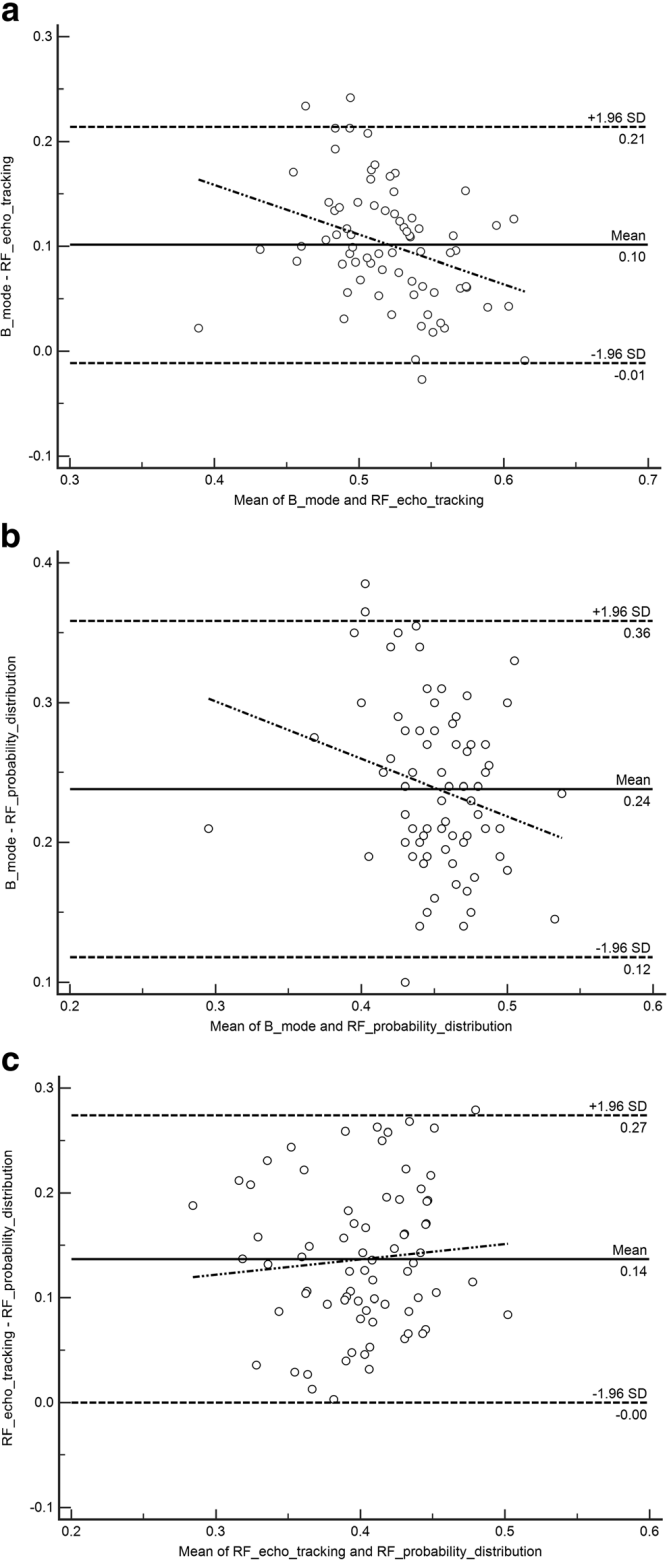
Bland-Altman scatter plots show agreement between the three techniques (*n*=74: *dashed lines* represent the 95% CI and the regression line). *Solid line* represents the mean difference. **a** Relationship between B-mode and radiofrequency echo tracking. The range of agreement was −0.01 mm to 0.21 mm; slope: -0.47 (*P*<0.01). **b** Relationship between B-mode and radiofrequency probability distribution. The range of agreement was 0.12 mm to 0.36 mm; slope: -0.41 (*P*=0.04). **c** Relationship between radiofrequency echo tracking and radiofrequency probability distribution. The range of agreement was 0.00 to 0.27 mm; slope: 0.14 (*P*=0.45). As the mean in (**a**) and (**b**) increases, the difference between the techniques decreases

The Wilcoxon test showed significant differences of 0.10 mm (95% CI: 0.09–0.11, *P*<0.001) for the comparison of the B-mode and RF echo tracking techniques, a difference of 0.24 mm (95% CI: 0.23–0.25, *P*<0.001) for the B-mode and RF speckle probability distribution techniques and a difference of 0.14 mm (95% CI: 0.12–0.16, *P*<0.001) for the comparison of the RF echo tracking and RF speckle probability distribution techniques. ICC for the comparison of all three techniques was 0.34 (95% CI: 0.27–0.39). Agreement and regression analysis between any two techniques is shown in the Bland-Altman scatter plots (Fig. 5).

The mean B-mode intima-media thickness for normal BMI group (*n*=59) was 0.55+/−0.006 mm (95% CI: 0.54–0.56). For the increased BMI group (*n*=55), B-mode intima-media thickness was 0.57+/−0.006 mm (95% CI: 0.56–0.58). The mean RF echo tracking intima-media thickness for the normal BMI group (*n*=46) was 0.46+/−0.008 mm (95% CI: 0.45–0.47) and for the increased BMI group (*n*=33), the mean was 0.49+/−0.008 (95% CI: 0.48–0.50). The mean RF speckle probability distribution intima-media thickness for the normal BMI group (*n*=60) was 0.32+/−0.006 mm (95% CI: 0.31–0.33). For the increased BMI group (*n*=60), the mean was 0.36+/−0.006 mm (95% CI: 0.35–0.37).

Intima-media thickness was statistically significantly higher in the increased BMI group for the RF echo tracking and the RF speckle probability distribution techniques and borderline significant for the B-mode technique. The mean differences using the Mann-Whitney *U* test were calculated at 0.02 mm+/−0.01 (95% CI: 0.00–0.04, *P*=0.05) with the B-mode technique, 0.03 mm +/−0.01 (95% CI: 0.01–0.05, *P*=0.02) with the RF echo tracking technique and 0.03 mm +/−0.01 (95% CI: 0.01–0.05, *P*=0.002) for the RF speckle probability distribution technique. Although age was statistically different between the groups, the difference was not clinically significant (11.4 and 12.0 years). Furthermore, the Pearson correlation did not show a significant correlation between intima-media thickness and age ranging between 0.04 (for B-mode and RF speckle probability distribution) to 0.15 (for RF echo tracking). The AEPC working group and Mannheim consensus stated that there are differences in the left and right intima-media thickness measurements being increased on the left side. We herein report an averaged right- and left-side intima-media thickness including one-side data for some participants who did not have both sides. Results remained unchanged when we included only the averaged data for the B-mode and RF speckle probability distribution (*P*=0.032 and *P*=0.005, respectively). However, the statistical significance was lost for the small sample of RF echo tracking (*P*=0.25).

## Discussion

Most of the literature on intima-media thickness measurement is on adults. In this study, we compared the various measurement techniques in children ages 10–13 years. We found that the three techniques yielded significantly different results. However, regardless of the technique used, the common carotid artery intima-media thickness seemed to increase in children with increased BMI compared to those with normal BMI.

Schreuder et al. [4] found a good correlation between B-mode and RF echo tracking techniques in adults with neurological symptoms related to cardiovascular diseases. They also found that B-mode values were higher than those measured by RF echo tracking, similar to our results [4]. Bianchini et al. [19] showed good agreement between B-mode automated system measurement and RF echo tracking techniques in adults with known cardiovascular disease risk factors and in healthy controls. The reported correlation between the two techniques in adults is between 0.76 and 0.86 [4, 20, 21]. These studies state that RF echo tracking-based measurements offer more precision than B-mode measurements. The Mannheim consensus included criteria for obtaining standardized intima-media thickness to facilitate data collection, interpretation and comparison [13]. The consensus also noted that manual measurements are more observer dependent than semiautomated measurements.

In children, manual and semiautomated measurements have been done using B-mode US. Some reports used RF echo tracking techniques [7]. These studies presented a normative range of measurements depending on age and gender [3, 6, 7, 22]. None has compared these techniques. The present study showed poor to fair correlation between techniques, but it also highlighted two important factors. First, it demonstrated that although very small, there is a statistically significant lower intima-media thickness in the normal BMI group of children as compared to the increased BMI group using the RF echo tracking and RF speckle probability distribution techniques. Second, the values we obtained for either B-mode or RF echo tracking were close to those published in the literature. Using semiautomated B-mode measurement, Bohm et al. [6] found a mean intima-media thickness of 0.51 mm for children around 10 years of age, which is close to the 0.56 mm observed in our cohort. Engelen et al. [7] found a thickness of 0.4 mm using RF echo tracking for 15 year olds, which is close to the 0.47 mm in our cohort. RF speckle probability distribution has the smallest values; however, this is close to the 0.38 mm of the cohort of Jourdan et al. [22] where a manual B-mode technique was used. The difference between the techniques could be related to the fact that the intima-media thickness is smaller in children than adults and therefore accurate measurement is challenging. This is in keeping with the observation that the regression graphs show a smaller difference with larger measurements.

Since intima-media thickness in pediatrics is sensitive to technique and dependent on age [23] and perhaps gender, the AEPC working group set standards for measuring and reporting it. This is a necessary step before drawing conclusions about intima-media thickness as a surrogate marker of subclinical atherosclerosis, especially in obesity [3]. In the same perspective, the American Heart Association published a scientific statement in 2009 recommending standardized assessment of intima-media thickness and arterial stiffness parameters in children [8]. The cited studies, as ours, demonstrated a relationship between intima-media thickness and BMI in children and between obesity in children and intima-media thickness in adulthood [8]. Many of these recommendations are in agreement with those of the AEPC working group in terms of technique used. Our results showed clear differences in intima-media thickness between normal BMI and increased BMI group for the RF techniques and a similar trend for B-mode. This is in agreement with the study published by Ozcetin et al. [24] using RF echo tracking. Intima-media thickness is therefore a potential marker of early vascular changes in at-risk children. However, it should be used in combination with other markers, such as pulse wave velocity or elastography in order to increase specificity. This observation applies to children [8, 25] and adults alike [26].

Our study has several limitations, including the small number of measurements due to the low success rate of the RF echo tracking technique. Since the measurements are semiautomated, interobserver variability becomes less significant. We did not study the interobserver variability due to the small error of estimating the 1-cm distance from the carotid bifurcation. Our measurements did not include different angles when acquiring the images, contrary to recommendations in the Mannheim consensus. Although we used the Mannheim consensus recommendations for image acquisition, we did not have ECG monitoring for B-mode based measurements. Selamet Tierney et al. [27] showed good reproducibility in intima-media thickness measures using either ECG-gated R-wave or subjective optimal visualization. However, values using R-wave were larger [27]. RF techniques gave an averaged measure over the entire cardiac cycle. Finally, we did not control for the arterial diameter for both groups and although we averaged left and right measurements, some participants did not have both sides screened. But Loizou et al. [28] did not show a difference between both sides in healthy asymptomatic subjects. This study does not allow us to say which technique is the gold standard.

## Conclusion

RF-based intima-media thickness in our study was increased in the overweight and obese children compared to normal weight children. This difference tends to be borderline significant for the B-mode technique. The success rate for the RF echo tracking automated technique is inferior to the other two techniques. The three techniques considered were not interchangeable suggesting that a single technique should be used in prospective studies across time for comparability.
